# Community health workers at the dawn of a new era: 8. Incentives and remuneration

**DOI:** 10.1186/s12961-021-00750-w

**Published:** 2021-10-12

**Authors:** Christopher J. Colvin, Steve Hodgins, Henry B. Perry

**Affiliations:** 1 Department of Public Health Sciences, University of Virginia, Charlottesville, VA USA; 2 School of Public Health, University of Alberta, Edmonton, AB Canada; 3 Health Systems Program, Department of International Health, Johns Hopkins Bloomberg School of Public Health, Baltimore, MD USA

**Keywords:** Community health workers, Incentives, Motivation, Compensation, Performance

## Abstract

**Background:**

This is the eighth in our series of 11 papers on “CHWs at the Dawn of a New Era”. Community health worker (CHW) incentives and remuneration are core issues that affect the performance of individual CHWs and the performance of the overall CHW programme. A better understanding of what motivates CHWs and a stronger awareness of the social justice dimensions of remuneration are essential in order to build stronger CHW programmes and to support the professionalization of the CHW workforce.

**Methods:**

We provide examples of incentives that have been provided to CHWs and identify factors that motivate and demotivate CHWs. We developed our findings in this paper by synthesizing the findings of a recent review of CHW motivation and incentives in a wide variety of CHW programmes with detailed case study data about CHW compensation and incentives in 29 national CHW programmes.

**Results:**

Incentives can be direct or indirect, and they can be complementary/demand-side incentives. Direct incentives can be financial or nonfinancial. Indirect incentives can be available through the health system or from the community, as can complementary, demand-side incentives. Motivation is sustained when CHWs feel they are a valued member of the health system and have a clear role and set of responsibilities within it. A sense of the “do-ability” of the CHW role is critical in maintaining CHW motivation. CHWs are best motivated by work that provides opportunities for personal growth and professional development, irrespective of the direct remuneration and technical skills obtained. Working and social relationships among CHWs themselves and between CHWs and other healthcare professionals and community members strongly shape CHW motivation.

**Conclusion:**

Our findings support the recent guidelines for CHWs released by WHO in 2018 that call for CHWs to receive a financial package that corresponds to their job demands, complexity, number of hours worked, training, and the roles they undertake. The guidelines also call for written agreements that specify the CHW’s role and responsibilities, working conditions, remuneration, and workers’ rights.

**Table:** 

Key message box 1. Summary	
Key findings	
We reviewed the literature to identify what kinds of incentives and remuneration have been offered to community health workers (CHWs), how these incentives have influenced CHW motivation, and how programmatic and contextual factors have shaped the relationships between incentives and CHW motivation • There is an extremely wide variety of incentives offered in CHW programmes around the world, and these function at individual as well as health system and community levels • CHW motivations can be affected by tensions between altruistic and material imperatives, by the social dimensions of their relationships to members of the health system and community, and by cultural, economic, and political contexts • CHW motivations are likely to change over time, which means that CHW programmes must be able to assess and respond to changes in the effectiveness of various incentives • WHO guidelines on CHWs have recently emphasized the importance of decent working conditions and fair labour practices in CHW programmes	
**Key implications**	
Our review concludes that: • Sustaining CHW motivation requires focused and consistent investment in locally meaningful and sustainable forms of incentives, typically with some clear support from or involvement from the state. CHW motivation cannot be sustained if CHWs are seen as a temporary or a civil society solution to health system failures • Sustaining CHW motivation requires thinking about incentives as multidimensional and about CHW motivation as something that changes over time • All of this in turn requires a health system that is functional and effective enough to provide a clear role and proper support for CHWs	

## Background

A perennial challenge in community health worker (CHW) programmes is the question of how to motivate people to engage in community health work as CHWs, to remain in these positions once trained, and to perform their work effectively over time. And, equally important, what kind of financial remuneration is fair, just, appropriate, and sustainable? Motivation is a complex phenomenon. For example, in the Introduction to this collection, Hodgins et al. [1] identify several design questions that have defined debates over how to best structure and implement CHW programmes. Are CHWs lackeys or liberators, lay or professional, members of government or civil society, generalists or specialists? When designing a CHW programme, the answers to each of these dilemmas will in turn determine the necessary strategies for developing and sustaining CHWs’ motivation to join programmes, remain in service, and perform effectively.

A wide variety of approaches have been used to motivate CHWs, but given the complexity of the task, there is still considerable uncertainty as to how to develop and sustain motivation in ways that are responsive to both more general psychological dynamics as well as the local social, cultural, economic, and political contexts in which CHW programmes operate [2–5]. The most common strategy for developing and sustaining motivation in CHW programmes centres on the use of discrete *incentives*, often framed in a fairly narrow fashion, as specific forms of reward—like payments, salaries, promotions, or awards for specific tasks or levels of performance. However, one can also define incentives as any factor that increases motivation to engage and perform well in CHW work. This more expansive model of incentives is rooted in the idea that the factors that motivate CHWs are far more numerous and complex than just the explicit financial or nonfinancial incentives offered by programmes.

Bhattacharyya et al. [6] provided the first systematic look at incentives and disincentives for CHW programmes and highlighted the complexity of CHW motivations: the importance of local context, the need for multiple incentives, the significance of community support, and the power of nonmonetary incentives. Since their review almost 20 years ago, global health interest in CHW programmes has only increased [7] as has the number of CHW programmes, largely driven by the evidence of effectiveness of community-based interventions for maternal and child health that CHWs can provide [8] and by the improvements in health made in a number of countries following the implementation of strong national CHW programmes. The most notable examples of these countries are Bangladesh, Brazil, Ethiopia, and Nepal [9]. The contributions that CHWs can make to the control of HIV/AIDS, malaria, and tuberculosis (TB) has also fuelled global interest in CHW programmes in southern Africa [10]. The question of how to motivate CHWs has thus only grown in scale and urgency.

This paper is the eighth paper in a series that provides a broad overview of issues and perspective on national CHW programmes in light of the growing importance for achieving universal health coverage, their affirmation for the first time by the World Health Assembly of their importance for improving population health, and the recognized need to build stronger integration of CHWs into national health systems [11]. This paper aims to update and extend the findings of Bhattacharyya’s review of CHW incentives by synthesizing recent evidence on the range of existing incentive schemes and strategies, developing an overarching conceptual framework for understanding how incentives work, and identifying a range of questions to be considered when designing CHW programmes that will motivate CHWs effectively. In the process, we also hope to deepen the theoretical discussion about how incentives translate into motivation and, in turn, into more effective practice. Context is, as always, crucial, and there should be widespread recognition that a one-size-fits-all approach is not sufficient [6]. This paper draws on the recently published compendium of 29 national CHW programmes throughout the world and the compendium’s findings related to incentives [9]. It also synthesizes findings from several previous reviews and case study projects addressing CHW motivation and incentives in a wide variety of CHW programmes that the authors have conducted. These prior projects include a realist review [12]^1^ from 2013 of the literature on incentives in CHW programmes in sub-Saharan Africa [13], a Reference Guide from 2014 for developing and strengthening CHW programmes [14], and a recently published book of case studies of national CHW programmes [9]. We have also integrated recent literature found in systematic reviews we have published as well as the extensive literature cited in the WHO 2018 guidelines for optimizing CHW programmes [15]. This paper aims to contribute, therefore, to a richer understanding of the mechanisms linking incentives, motivation, and performance and the role of context in shaping these mechanisms. We recognize that the paper provides a stronger focus on motivation among volunteer CHWs and would be strengthened by a stronger emphasis on salaried CHWs.

## Examples of CHW incentives

**Table:** 

Key message box 2	
The literature reports an extremely wide variety of incentives used in CHW programmes to create and sustain motivation	

In this section, we survey some examples of the kinds of incentives reported in a recent systematic review by Scott et al. [16] prepared to support the production of the WHO guidelines for CHW programmes that were released in 2018 [15].

A number of studies reported that CHWs had significant complaints about incentives. A review of 81 studies of intervention design factors that influence performance [17] found that in almost one third (31%), CHWs were dissatisfied with their incentives, and the level of satisfaction (or dissatisfaction) with incentives was closely linked to CHW motivation and performance (or lack thereof). There is evidence that improving financial remuneration can reduce attrition [18, 19]. One recent report argued that reliance on performance-based incentives alone does not provide CHWs with financial security and may ultimately impede CHW rights [20]. One systematic review of papers that explored the determinants of success in scaling up and sustaining CHW programmes in low- and middle-income countries noted that the most frequently identified barrier to scale-up and sustainability of CHW programmes was insufficient pay/incentives, cited in eight of the 19 articles in the review [18]. A study of health surveillance assistants in Malawi found that a lack of financial incentives and shortages of basic supplies and materials were key demotivation factors [21].

Unmet promises related to incentives and remuneration, delayed release of payments, and having to spend out of pocket to meet one’s responsibilities were identified as sources of demotivation for CHWs [22]. From the community perspective, salaried CHWs could be seen as less motivated by altruistic motives than by financial motives, and communities may see salaried CHWs as primarily health system functionaries rather than equally accountable to the community [23].

Ormel et al. [24] undertook a comparative analysis of qualitative studies of CHW motivation and performance in six countries (Bangladesh, Ethiopia Kenya, Indonesia, Malawi, and Mozambique). They concluded that intrinsic rewards are important for both volunteer and salaried CHWs, but they do not compensate for the demotivation produced by a perceived low level of financial reward. While introducing financial incentives is motivating, adequate expectation management is needed to prevent frustration as a result of broken promises either as a result of failure to provide them or delays in providing them.

CHW volunteers in Ghana expressed a need for raincoats and strongly desired identifiers such as T-shirts and certificates as something tangible to show that their work is recognized and appreciated [25]. A discrete choice experiment with 199 CHW volunteers in western Kenya was designed to measure the relative importance of different incentives [26]. The respondents’ most highly valued incentive was “high levels of community appreciation for their work” which was valued the same as a $20-per-month transport allowance, and valued more highly than appreciation from health facility staff or trainings six times per year. Ethnographic research together with focus group discussions and in-depth key informant interviews in rural Uganda revealed that community members are willing to reward volunteer CHWs (who are members of family health teams) with cash as well as material and symbolic rewards for appreciation of their help. Motivating factors that would encourage the community to provide these rewards included recognition by the medical staff of the health system of the importance of the CHWs, fulfilment of promises made to the community by the government, and exemplary behaviour by CHWs [27]. A realist evaluation of the types of activities in which the Red Cross in Uganda could engage to influence the motivation and performance of volunteer CHWs drew on participant observation and interviews, and concluded that the most effective activities would be (1) supervision supportive of autonomy and (2) skills and knowledge enhancement. This would lead to satisfaction of the three key drivers for volunteer motivation: (1) feelings of autonomy, (2) competence, and (3) connectedness and would presumably lead to higher retention, better task performance, and well-being among the volunteer CHWs [28].

CHW volunteers in Nepal and Bhutan feel a deep sense of satisfaction from serving their community on a voluntary basis, and they feel that the rewards of performing their role in terms not only of personal satisfaction but also of appreciation by the community is fully sufficient without any financial remuneration [29]. Female community health volunteers (FCHV) in Nepal “appear to be influenced by a tradition of volunteering as moral behaviour, a lack of respect for paid government workers, and the [FCHV] Programme's community embeddedness” [29]. In Bhutan, volunteer CHWs place high value on the motivation and encouragement they receive from family and their spouse, and from the community feeling of being grateful for the services provided by the CHW [30].

However, some have argued that volunteer CHW work is by definition unfair and unjust and should be reimbursed appropriately with a living wage along with a seat at the table in decisions about health systems strengthening. To not do so is exploitation, especially when CHWs are working in externally funded disease-control programmes such as the HIV/AIDS programme in Ethiopia [31]. This is partly because volunteer CHWs themselves are most commonly women and often desperate because of unemployment and food insecurity. Many programmes provide per diems, but the small amounts provided raise questions about “labor rights and justice in global health pay structures” [32]. One recently reported study of CHWs in Madagascar found that among 874 unpaid CHWs across the country, 92% were food-insecure, and 89% had experienced a shock in food household security in the previous year. Eighteen percent reported that they had used some of their personal financial resources to continue their work [33]. The authors concluded that programme managers need to take into account the precarious financial situation of CHWs who are volunteering their time.

Abdel-All and colleagues carried out a discrete choice experiment with 318 accredited social health activist (ASHA) workers in south India to understand their preferences for jobs that provide various types of incentive and remuneration options and how much of their salary they would be willing to forgo in order to obtain these benefits [34]. There was heterogeneity among the respondents, with one group favouring jobs that provided training leading to a promotion, a fixed salary, and free family healthcare (51% of the respondents), another group favouring free family healthcare (35% of the respondents), and a final group giving preference to a more reasonable workload (13% of the respondents).

Bernal and Martinez randomly assigned groups of CHWs in Honduras into intervention and controls groups, and the intervention group received team incentives based on their team’s performance in achieving standard population-based performance targets for maternal and child health services based on household surveys [35]. Every 6 months, teams in the intervention area were able to select awards based on their performance, including laptops, air conditioners, microwaves, and other items that could be used by their health unit. The value of the award was based on the performance targets reached. After 12 months, the outcome indicators in the intervention area were significantly greater than in the control area for community outreach, quality of care, timeliness of care, and utilization of maternal and childcare services.

Ballard and colleagues have carried out an assessment of the legal frameworks for CHW compensation in five countries: Brazil, Ghana, Nigeria, Rwanda, and South Africa [36]. They propose that each country represents a distinct archetype for compensation: public sector employment (Brazil, community health agents), private sector employment (Nigeria, CHWs), hybrid public/private (South Africa, CHWs), cooperatives with performance-based incentives (Rwanda, CHWs), and volunteer-based (Ghana, community health volunteers). The authors conclude that the public sector employment model that Brazil has established provides the best option for institutionalizing the WHO recommendations for formal contractual employment with paid CHWs that meet the international standards for fair and just work. Stronger political will, long-term financing, and the passing of appropriate national legislation will be required to attain the cadres of paid, professionalized CHWs that will be needed for CHW programmes to reach their full potential.

A recent policy paper from the World Bank calls for a new approach by governments to the support of CHWs in the face of the COVID-19 pandemic [37]. Using India as a point of reference, the authors argue that during this time of unprecedented crisis, building trust in public institutions is urgently needed for public health, “and the forces of change in local politics and health bureaucracies … can be harnessed by policy-makers to establish a cadre of trustworthy and professional frontline public health workers” [p. 34]. The paper calls for “a leap of faith in recruiting, training, empowering and equipping frontline health workers [ASHA and Anganwadi workers] with steady wages. Investing in this cadre to manage and prevent disease outbreaks in their communities, liaising with the higher-tier health system as advised to do so by the global scientific and technical research community, is indispensable” [p. 35].

## Recent findings on incentives and reimbursement in national CHW programmes

**Table:** 

Key message box 3	
Salary and non-salary incentives are often the object of concern and even collective action among CHWs in national CHW programmes	

While the incentive strategies and motivational approaches of most CHW programmes are not well described in the grey or academic literatures, one area where we have been able to develop a more concrete and coherent picture of practice across a diverse mix of settings is salaries and other forms of remuneration. Here we provide a more detailed section on types of salary packages and conditions of service offered.

A recently released book containing case studies of 29 national CHW programmes provides an up-to-date picture of the incentives and reimbursement [9]. The low level of compensation for CHWs was the third most frequently mentioned challenge for the programmes, after (1) lack of supplies and (2) inadequate, unstable, or unsustainable financing. Common themes across the case studies are (1) a feeling among CHWs that they are underpaid and that their remuneration inadequately reflects the time and effort they contribute, (2) their pay arrives irregularly and is sometimes delayed by months, and (3) expenses they incur are not appropriately reimbursed. Increasingly, CHWs are taking collective actions to demonstrate their frustrations and to present their demands before legislative bodies and ministries of health. This has happened in Bangladesh, India, South Africa, and Pakistan. Table 1 provides information about each of the national programmes, whether they are formally employed or not, work full-time or part-time, their salary (if any), other types of incentives received, and any additional compensation.

**Table 1:** Compensation and incentives provided to CHWs in national programmes

Country	CHW cadre	Formal employee vs volunteer	Full-time vs part-time	Salary	Other types of support and incentives	Additional compensation
Afghanistan	Community Health Worker (CHW)	Volunteer	Part-time	Unpaid	Allowances to cover travel and food for all monthly meetings at the facility and any training courses they attend The TB programme offers two prizes of US$ 100 per province per quarter to the best-performing health workers	Many communities have built a house or an extension to the CHW’s house for the health post, assist with transport, promote health campaigns, and support the CHWs in their work with individual families
Bangladesh	*Shasthya Shebika* (BRAC)	Volunteer	Part-time	Unpaid	NA	Small loans to establish revolving funds, which they use to make some money by selling health products at a small mark-up Monthly performance-based incentives
	*Shasthya Kormi* (BRAC)	Formal employee	Part-time	$190 per month	NA	NA
	Family welfare assistant (FWA) (government)	Formal employee	Full-time	$132–$318 per month	NA	NA
	Health assistant (HA) (government)	Formal employee	Full-time	$132–$327 per month	NA	NA
	Community healthcare provider (CHCP) (government)	Formal employee	Full-time	$150–$362 per month	NA	NA
Brazil	*Agente Comunitário de Saúde* (ACS)	Formal employee	Full-time	$281–$472 per month	NA	NA
Ethiopia	Health extension worker (HEW)	Formal employee	Full-time	$86 per month	Opportunities for career advancement	NA
	Women’s Development Army (WDA)	Volunteer	Part-time	Unpaid	Formal recognition from the health system Ongoing mentorship by HEWs Certificates Recognition by the community and local leaders	NA
Ghana	Community-based Health and Planning Services (CHPS) community health officer (CHO)	Formal employee	Full-time	$140 per month	NA	Extra paid leave days The opportunity to advance their education with paid education leave
	CHPS community health volunteer (CHV)	Volunteer	Part-time	Unpaid	T-shirt, transport per diem, and bicycle	NA
Guatemala	Promotor de salud*	NA	NA	NA	NA	NA
	Guardian de Salud*	Formal employee	Full-time	$50 per month	Community recognition and family support	NA
India	Auxiliary nurse-midwife (ANM)	Formal employee	Full-time	$280 per month	NA	NA
	Anganwadi worker (AWW)	Volunteer	Full-time	$50–$130 per month	NA	NA
	Accredited social health activist (ASHA)	Volunteer	Part-time	$42–$56 per month	NA	NA
	Village health guide (VHG)*	Volunteer	Part-time	$24 per month	NA	NA
Indonesia	*Kader*	Volunteer	Part-time	Unpaid	May receive informal types of compensation, such as free medical treatment from higher levels in the health system	NA
Iran	*Behvarz* (rural)	Formal employee	Full-time	$350 per month	NA	Adjustments are made based on a combination of catchment area size and performance
	*Moraghebe-Salamat* (urban)	Formal employees	Full-time	$350 per month	NA	Adjustments are made based on a combination of catchment area size and performance
Kenya	Community health volunteer (CHV)	Volunteer	Part-time	$20–$60 per month	NA	NA
Liberia	National community health assistant (CHA)	Formal employee	Part-time	$70 per month	Training certificate, formal role in the public health system, commodities, and medical supplies	NA
	Community health service supervisor (CHSS)	Formal employee	Full-time	$313 per month	NA	Gasoline allotment
Madagascar	*Agent Communautaire* (AC)	Volunteer	Part-time	Unpaid	Travel, equipment and materials, food, community recognition and support, promotion to a supervisory position	Per diems for trainings and meetings when there is a training or campaign Indemnities Income from user fees Access to savings and lending groups and insurance groups Support for income-generating activities
	*Agent Communautaire de Nutrition* (ACN)	Volunteer	Part-time	Unpaid	Training, travel opportunities, equipment and materials, food, community recognition and support, promotion to a supervisory position	$17 per month when there is an active project Per diems for trainings and meetings when there is a training or campaign Indemnities Income from user fees Access to savings and lending groups and insurance groups Support for income-generating activities
Malawi	CHW	Formal employees	Full-time	$63 per month	Uniform, T-shirt, bag, public recognition and a bicycle	Per diems
Mozambique	*Agentes Polivalente Elementare* (APE)	Volunteer	Part-time	$20 per month	Bicycle, flashlight, vest, medicine bag, identification badge, hat, calculator, thermometer, and stopwatch (to measure respiratory rate)	NA
Myanmar	Auxiliary midwife (AMW)	NA	NA	NA	Service packages, refresher training, continuous supplies, regular supportive supervision, as well as recognition and career advancement opportunities	NA
	CHW	NA	NA	NA	NA	NA
	Malaria volunteer	Volunteers	NA	Quarterly incentive, amount unknown	NA	NA
	TB volunteer	Volunteers	NA	A set amount per TB case referred	NA	NA
	Traditional birth attendant	NA	NA	NA	NA	NA
Nepal	Female community health volunteer (FCHV)	Volunteer	Part-time	Unpaid	Refresher trainings, a uniform, a bag and a start-up kit of supplies	Training allowances, an annual clothing allowance, access to microcredit funds
Niger	*Agent de Santé Communautaire* (ASC)	Formal employee	Full-time	$100 per month	NA	NA
	*Relais* volunteer	Volunteer	Part-time	Unpaid	Gifts of produce from crops	$20 per month, but this is irregular and infrequent Occasional payments for trainings or participation in mass campaigns
Nigeria	Community health extension worker (CHEW)	Formal employee	Full-time	$281 per month	NA	NA
	Voluntary village health worker (VVHW) and community-directed distributor (CDD)	Volunteer	Part-time	Unpaid	NA	NA
Pakistan	Lady health worker (LHW)	Formal employee	Full-time	$85 per month	NA	NA
Rwanda	*Binôme*	Volunteer	Part-time	Unpaid	NA	Performance-based financing incentives One is for achievement of specified targets for the CHW cooperative (and the cooperative receive the incentive) Another is for individual event-based performance
	*Agent de Sante Maternelle* (ASM)	Volunteer	Part-time	Unpaid	NA	
Sierra Leone	National CHW	Volunteer	Part-time	CHW: $14 per month Peer supervisor: $20 per month	T-shirt, backpack, and supplies	CHWs: Those CHWs serving communities within 3 km of a health facility receive an additional $7 per month Those CHWs serving communities 3 km or further from a health facility receive an additional $11 per month Peer supervisors: Receive an additional $14 for transportation and logistics
South Africa	Ward-based primary healthcare outreach team (WBPHCOT)	Volunteer	Part-time	$150–$290 per month	NA	NA
Tanzania	CHW	Formal employee	Full-time	$143 per month	Community recognition	NA
Thailand	Village health volunteer (VHV)	Volunteer	Part-time	$20 per month	Sponsored leisure activities, parties, and field trips	Possible national awards
Zambia	Community health assistants (CHA)	Formal employee	Full-time	$390 per month	A bicycle, all-weather boots, a backpack, and a uniform	NA
Zimbabwe	Community-based volunteer (CBV)	Volunteers	Part-time	Unpaid	NA	NA

*NA* not available

Incentives and remuneration vary considerably. Many programmes utilize volunteers and provide no salary (Afghanistan, Ethiopia, Indonesia, Kenya, Nepal), but these volunteers usually receive other incentives, from per diem payments to social recognition and in-kind benefits. Thailand is remarkable because of its commitment and capacity to pay such a large number (1 million) of village health volunteers (that is, one for every 40–80 people) a regular stipend of US$ 20 per month along with other nonfinancial incentives [38]. And India is also remarkable for paying its 1 million ASHA workers US$ 42–56 per month along with additional performance incentives together with its 1.3 million Anganwadi workers (who receive US$ 50–130 per month) and 0.2 million auxiliary nurse midwives/multipurpose health workers, who receive US$ 280 per month [39]. As shown in Table 1, most of the programmes documented in this compendium provide payments to full-time CHWs that are typically in the range of US$ 100–300 per month. The most highly paid CHWs in the review are Nigeria’s community health extension workers (CHEWs) who receive US$ 281 per month and Iran’s *Behvarzs* who receive US$ 350 per month.

Many programmes provide their CHWs with a monthly payment but call this an *incentive* rather than a salary that a regularly employed person would receive. As such, this income lacks the associated benefits that government employees normally receive. Ethiopia, Ghana, Malawi, and Nigeria are notable exceptions, since their higher-level CHWs are all in formal civil service positions.

The Bangladesh Rural Advancement Committee (BRAC) CHW programme is an interesting outlier in several respects, including payment. It has one of the largest nongovernmental cadres of CHWs in the world. BRAC has a dual-cadre CHW system in which the lower level of CHWs, called *Shasthya Shebikas*, make a commission on sales of health-related products. These CHWs are also members of women’s savings and loan groups called voluntary organizations, whose other members engage in a variety of income-generating activities such as raising chickens, producing milk, and making handicrafts. This approach has made it possible for the CHW programme to grow without dependence on external funding [40].

Performance-based financing has now entered the realm of payments to CHWs in a number of countries. India’s ASHAs receive performance-based bonuses for referring women to deliver at a facility [39]. Iran’s *Behvarzs* receive performance-based incentives [41]. Payments to Rwanda’s CHWs are partly based on performance related to indicators for nutrition, antenatal care, facility deliveries, family planning services, and engagement with HIV and TB control [42]. Thirty percent of the payment goes to individual CHWs, and the remaining 70% to the cooperative. Among other things, this approach encourages teamwork as well as high individual performance [42].

It is important to note that the recently released WHO guidelines for CHW programmes [15, 43] specifically recommend *against* paying CHWs exclusively or predominantly using performance-based incentives. The unintended consequence of relying too heavily on performance-based incentives is that CHWs focus too much on the activities that contribute to the measurement of the performance indicator at the expense of other important activities.

A variety of other forms of provision of incentives and non-salary financial payments are currently in use by these 29 national CHW programmes. Table 2 summarizes some of these other forms of non-salary incentives.

**Table 2:** Non-salary incentives for CHWs

Incentive	Commentary
Assistance with the formation or functioning of CHW groups	In a number of countries, national CHW programmes assist CHWs with the formation of local groups of CHWs which can serve a variety of different purposes. In Rwanda, CHW cooperatives have been established. These cooperatives receive funds directly from the government for the services of its members, and the members work together using these funds as seed money for other income-generating activities [42]. In Madagascar, CHWs have access to savings and lending groups and insurance groups [44]. In Nepal, female community health volunteers have access to microcredit groups and loans. In Nepal, the government has also established local endowment funds for local endowment funds for FCHVS throughout the country. Female community health volunteers are able to draw loans to pursue income-generating activities or receive small payments for their work [45]
Supplies and equipment required for the work	Having the needed supplies and equipment are the basis for good performance, of course, but unfortunately, CHWs are often lacking essentials. Having what is needed is a source of affirmation and motivation. Examples of such items that programmes provide include uniforms, identification badges, T-shirts, hats, all-weather boots, backpacks, bicycles, start-up kits of supplies, flashlights, thermometers, and stopwatches (to measure respiratory rate). Occasionally, gasoline is provided as in the case of community health services supervisors in Liberia [46]
Per diem payments for work	In many CHW programmes, particularly for those that use volunteers or for programmes that are funded by disease-control programmes, CHWs receive a per diem payment to participate in special campaigns for community mobilization activities, such as immunization days, child health days, and so forth
Job-related nonfinancial benefits	In Afghanistan and Ghana, communities assist with housing for CHWs or with the construction of a health post as an extension of the house [47, 48]. In Indonesia, *Kaders*, who are volunteers, receive free medical care from higher levels of the health system [49]. Per diem payments for trainings are often attractive to CHWs since they not uncommonly provide some additional income beyond the actual costs involved in attending a training
Career advancement	Opportunities for career advancement and further educational opportunities are highly valued by CHWs. In some cases (e.g., in Ethiopia), a health extension worker can advance to a higher pay grade after obtaining further in-service training, though they continue in their same role as before. They can also receive preferential consideration when applying to enter a training programme for a high-level health worker position [50]. In some programmes, CHWs can advance to a CHW supervisory position (as in Madagascar, Malawi, and Sierra Leone). Ghana provides its community health officers with paid educational leave
Public recognition for service	Many programmes give CHWs, especially those who volunteer, certificates or other forms of special recognition. Communities often provide recognition as well, including community labour to assist CHWs with their agricultural or construction work. Thailand provides its CHW volunteers with leisure activities, parties, and field trips [38]. Cash prizes for high performance are provided in Afghanistan [47]. In Niger, communities provide their *Relais* volunteers with gifts of produce from their crops [51]

## A typology of CHW incentives

**Table:** 

Key message box 4	
While direct financial incentives might be the most familiar form of incentive, there are many other kinds of incentives, operating at individual, health system, and community levels	

Table 3 lists numerous examples of CHW incentives that have been described in the literature and organizes these incentives into a conceptual model that delineates the different types and levels of incentives.

**Table 3:** Types of CHW incentives

Financial incentives	Nonfinancial incentives
Direct incentives	
Terms and conditions of employment: salary/stipend, pension, insurance, allowances, leave	Job satisfaction/work environment: autonomy, role clarity, supportive/facilitative supervision, manageable workload
Performance payments: performance-linked bonuses or incentives	Preferential access to services: healthcare, housing, education
Other financial support: reimbursement of costs (travel, airtime), fellowships, loans, ad hoc	Professional development: continuing training, effective supervision, study leave, career path that enables promotion and moving into new roles
	Formal recognition: by colleagues, health system, community, wider society
	Informal recognition: T-shirts, name tags, access to supplies/equipment, bicycles, etc.

Health system	Community-level
Indirect incentives	
Well-functioning health systems: effective management, consistent M&E, prompt monthly payments, safe environment, adequate supplies, and working equipment	Community involvement in CHW selection and training
Sustainable health systems: sustainable financing, job security	Community organizations that support CHWs
Responsive health systems: trust, transparency, fairness, consistency	CHWs witnessing visible improvements in health of community members
Complementary/demand-side incentives	
Healthcare workers witnessing and grateful for visible improvements in health of community members made by CHWs	Community members witnessing and grateful for visible improvements in health of its members
Policies and legislation that support CHWs	Successful referrals to health facilities
Funding for CHW activities from state or communities	CHW associations

CHW incentives are most commonly divided into *financial* and *nonfinancial* incentives. Both of these incentives can be understood as *direct incentives* since they are specific incentives offered directly to individual CHWs as part of a CHW programme (the first set of rows in Table 3). Most programmes offer some form of financial incentive. In larger, government-run programmes (the focus of this series of papers), these might be modest but full-time salaries. In nongovernmental organization (NGO)-run or community-supported programmes, these incentives might be small stipends and reimbursements for travel or airtime for cell phones. Rwanda and India, for example, have developed performance-based incentive programmes that reward CHWs for completion of certain tasks [9]. India also offers a life insurance programme to some of its government CHWs, and some NGO programmes in South Africa offer scholarships for further training. Common nonfinancial incentives found globally include formal uniforms, T-shirts, and name tags; access to bicycles and medical supplies; and preferential access to health or housing resources, largely in NGO-run programmes [9].

The second set of rows in Table 3 describes *indirect incentives*. Dambisya [52] uses this term to describe incentives that are “not specific to individuals or groups, but to the [health] system as a whole” (p. 5). In their typology of incentives and disincentives, Bhattacharyya et al. [6] also describe indirect incentives that operate within the community context and work to motivate individual CHWs. Health systems-related indirect incentives, such as good management, sustainable financing, fairness, and transparency have been identified by CHWs and programme managers alike as critical success factors for effective CHW programmes [22]. Indirect incentives at the community level include community involvement in CHW training and selection, and community support for the work of CHWs. These forms of community involvement are not intended to directly incentivize CHWs, but promoting a positive and effective working relationship with the communities they serve can be a powerful motivating force for CHWs [16].

Finally, incentives, whether direct or indirect, are generally conceived of with respect to their impact on the motivation of individual CHWs. Bhattacharyya et al. [6], however, make an important distinction in their typology between factors that motivate individual CHWs and factors that motivate others to support and sustain CHWs in general. We have labelled these *complementary incentives* to capture the ways they complement efforts to incentivize CHWs themselves. One example might be the greater support for CHWs and their work that can emerge when healthcare workers or community members witness tangible changes in health outcomes that are the result of CHW initiatives [16]. As with indirect incentives, we have divided these into health systems and community levels (see the third set of rows in Table 3).

This conceptual framework provides a useful way to compare and contrast the many kinds of incentives for CHWs that are possible. All of the literature we reviewed for this paper made reference to one or more of these types of incentives, and much of the literature is concerned with the interactions among these various types of incentives and their impact on CHW performance. Unfortunately, very little of the literature provides detailed explanations of how exactly incentives are designed and delivered, or what impact specific incentives might have had on CHW performance (typically focusing instead on their perceived impact on the intermediate step of motivation). When changes in performance outcomes were reported, they were typically CHW or programme manager perceptions of change [53]. However, there are a growing number of studies that provide a more rigorous assessment of the outcomes of CHW incentives strategies [17, 35, 54–58].

## Incentives and CHW motivation at the individual level

**Table:** 

Key message box 5	
Managing the relationship between material and altruistic motivations among CHWs is an important but complex and delicate balance to strike	

One of the most frequent debates about CHW incentives centres on the question of whether CHWs should be seen as *workers* (paid) or as *volunteers* (unpaid) [6, 59, 60], driven by, respectively, material or altruistic motives, and thus properly incentivized through either financial or nonfinancial means. Implicit in much of this discourse is an assumption that there are two distinct CHW models available to us, ones underpinned by two distinct and incompatible sets of motivations. The literature, however, reveals a much more complex picture (see Table 4, Finding 3.1). In most studies and contexts, for example, CHW motivation is linked, by CHWs themselves and by other programme staff, to a rich array of altruistic goals and intentions, often tied to religious or cultural frameworks [59]. It is also clear, however, that in most studies and contexts, CHWs generally come from the same, often poor communities they serve and see CHW work partly as a means of advancing both themselves and their families economically [61–63]. For many CHWs, this can pose a cultural problem since personal gain may be seen as incompatible with more altruistic motives [29], and they may choose to downplay these personal motives. Given the resource-constrained settings in which most CHWs live and work, however, it should be accepted as inevitable that CHWs will generally bring a mix of both material and altruistic intentions to their work.

**Table 4:** Incentives and CHW motivation at the individual level

Findings	Policy and management prompts
4.1 The coexistence of both altruism and the need to meet basic survival needs is an unavoidable aspect of the CHW motivation	How do CHWs describe the source of the altruism that keeps them engaged in their work? Is it a desire to be helpful to the community and neighbours in need? Is it religion? Cultural norms? Empathy for the suffering of others? Something else? How widely shared are these sources of altruism in the community? How does the CHW programme reinforce or undermine these sources of altruism? How do CHWs, programme managers, community members, or health facility staff perceive the relationship between altruism and personal benefit? How do CHWs try to manage any potential/perceived conflict? How can the incentives offered by the programme help them to sustain this balance?
4.2 Intrinsic incentives alone are not sustainable over time but can be paired with effective extrinsic (material) incentives, which can take multiple forms	What are the economic needs and challenges for CHWs? What other kinds of work are available to them? Besides a salary or stipend, how else might the programme help to address some of their other material needs (training and certification opportunities, referrals for/preferential access to health, welfare, housing, or educational benefits, food parcels, transport money, airtime, etc.)? What ways of distributing these material benefits among the CHWs would be socially acceptable, avoid breeding competition, and not challenge altruistic motives? Seniority in the programme? Need? Level of performance? Something else?
4.3 Though not sufficient by itself, intrinsic reward nonetheless often triggers CHW involvement and can be a necessary part of maintaining CHW motivation over time	Do the CHWs recruited for a programme have some personal connection to the health problem addressed or some other altruistic motivation for working as CHWs? What was their trigger for getting involved? Have they developed intrinsic motivations over time?

There is evidence in the literature that incentives that are linked primarily to altruistic motivations lead to high rates of attrition over the long term [61, 64], although there are certainly exceptions, as with the FCHVs in Nepal [45]. This does not mean, however, that all CHWs must be made into salaried employees and have their financial needs fully met to sustain their engagement. Rather, the material needs of CHWs can be addressed in a wide variety of ways, including some combination of salary, stipends, bonuses, and reimbursements along with nonfinancial incentives (commonly provided in NGO CHW programmes) such as uniforms, T-shirts, training opportunities, community recognition, preferred access to healthcare services, or access to bicycles. What seems to matter to CHWs is a sense that their material concerns are both recognized *and* mitigated by CHW programmes rather than completely resolved [65] (see Finding 4.2). Even stipends that are below the minimum or average wage in a community are often meaningful enough to keep CHWs, who might otherwise be completely unemployed, engaged in this work. Whether or not these stipends can be justified ethically or whether they are legal with respect to local labour law is a separate but important concern [66]. It is worth considering, however, that in virtually all low- and middle-income countries, the majority of the working-age population is engaged in informal-sector employment which, in general, does not come under effective government regulation. The recently released WHO guidelines for CHW programmes (discussed earlier) address this issue in some detail [15].

For many CHWs, altruism remains a critical factor in their motivation [67]. Many CHWs, for example, reported a personal experience with a health problem, either themselves or in their family, as a triggering event for getting involved in CHW work. Seeing the health and social benefits of their work in their family and community also serves as a critical way of sustaining motivation over the longer term [68–70]. Maintaining a connection to these altruistic principles is cited by many of these studies as a key factor in maintaining good job satisfaction and performance (see Finding 4.3). In volunteer CHW programmes that only require occasional participation (such as in child health days or distribution of bed nets), financial incentives may also play a more limited role in motivation than altruistic considerations.

## CHW incentives and motivation in health system and community context

**Table:** 

Key message box 6	
The relationships that a CHW has with other CHWs, with other healthcare workers, with managers, and with community members and leaders have a significant impact on motivation	

In addition to individual material or altruistic motives, CHW motivation is also affected by forces that lie beyond the individual. Whether framed as health *volunteers* or health *workers* or something in between, CHWs are, for example, almost always perceived as part of the health system and/or community in some way. The studies we reviewed catalogued in detail the ways in which health system and community environments can incentivize or disincentivize CHWs. Rather than summarizing all of the features of the local health system and community context that impact on CHW motivation, however, we have synthesized this evidence and describe below some of the broader principles at work in the ways health systems and community contexts promote or hinder CHW motivation. Paper 9 in this series addresses in more detail issues of engagement of CHWs with communities and the health system [71].

Our first key finding in this context is that CHW motivation is sustained when CHWs feel they are valued and respected members of the health system and have a clear role and set of responsibilities within it (see Table 5, Finding 5.1). A sense of being valued can be reinforced in a wide variety of ways. Some studies argue for the importance of formal modes of recognition both within the health system and in the community, including awards, certificates, and appreciation days [61, 65, 72]. Every day, informal signs of respect are equally important, though. These are the most direct and public ways of recognizing the value of CHWs and would seem to be the easiest to put in place, but also appear to be only weakly integrated into many programmes.

**Table 5:** CHWs in the system: findings on incentives within the health system

Findings	Policy and management prompts
5.1 CHW motivation is sustained when they feel they are a valued member of the health system and have a clear role and set of responsibilities within it	In what ways are CHWs recognized for their contributions to the health system? Do they have the chance to get positive feedback from other staff or from managers on a day-to-day basis? Are there more formal opportunities for recognition from members of the health system? Do CHWs have clear job descriptions and distinct roles in the settings where they work? Have their roles and responsibilities been communicated to the other healthcare workers with whom they work? Are there areas of ambiguity in their roles, or overlap with the roles of other workers?
5.2 A sense of the “do-ability” of the CHW role is critical in maintaining CHW motivation	How is the CHW’s set of responsibilities decided on and how do managers ensure CHWs have the capacity to fulfil these responsibilities? How will managers know if the workload or the requirements of the job are exceeding the capacities of individual CHWs? Do CHWs have the opportunity to speak about issues of workload or technical complexity? How are these issues managed? Do CHWs or local programme managers have the flexibility to reorganize tasks in ways that improve the “do-ability” of the role?
5.3 CHWs are best motivated by work that provides opportunities for personal growth and professional development, irrespective of the direct material and technical skills	What elements of the CHW role promote personal growth (e.g., social, emotional, psychological, or intellectual development)? How do CHWs understand and value the place of personal growth in their work? How can these elements be strengthened in the programme? What elements of the CHW role promote basic professional development (e.g., computer, administrative, financial, or logistical skills)? How do CHWs understand and value the place of this professional development in their work? How can these elements be strengthened in the programme?
5.4 Working and social relationships among CHWs themselves and between CHWs and other healthcare professionals and community members strongly shape CHW motivation	Do CHWs ever get the chance to work with or interact with each other in their daily work? If they work alone, are there other opportunities for them to engage with other CHWs? Are there formal or informal opportunities for CHWs to spend time with each other outside of the context of daily work? Are there CHW associations or networks? Do they even get the chance to meet CHWs working in other districts/regions to share experiences? What are the relationships like between the CHWs and healthcare professionals in your context? Does the work environment promote positive relationships? Are there conflicts between CHWs and other healthcare providers, and if so, how can these be addressed?
5.5 Relationships of recognition and accountability with the health system, community, and other stakeholders are multiple and complex and also affect CHW motivation	Are there processes for recognizing service and dedication among CHWs by the health system, the community at large, or other stakeholders? Are there issues of mistrust or antagonism of CHWs with the community or with other higher-level health workers? How are conflicts or issues of poor performance among CHWs handled and by whom? Are there multiple or confusing lines of accountability for CHWs (e.g., to both the health system and the community)? If so, how can these be clarified or reconciled?
5.6 Incentives operate within a social system and can affect these relationships as well	How are incentives offered to CHWs interacting with the existing social relationships among CHWs and between CHWs and others? Are the incentives exacerbating internal differences or hierarchies? Or, are they helping to build solidarity and common purpose among CHWs? Are incentives introducing new dynamics into these social relationships such as competition, status conflicts, or other forms of division? How can incentives be designed and managed in a way that limits any negative effects on these social relationships?

There are numerous other ways, however, in which the value of CHWs, or their lack of value, is implicitly communicated by the health system. The most damaging one reported is the withdrawal or inconsistent payment of stipends and other forms of material support [24, 34]. This was a powerful demotivator of CHWs, not so much for the ways it deepened material deprivation but for the message it sent to CHWs that they were not valued by the health system or community [73, 74]. Lack of adequate training, poor supervision and support, inadequate supplies and equipment or frequent stockouts, insufficient transportation, and other infrastructural deficits were other common disincentives that communicated the message that CHWs were not valued members of the health system or their community [72, 73, 75, 76].

Another critical source of CHW motivation is a sense of the “do-ability” [69] of the CHW role (see Finding 5.2). The kinds of problems described above not only made CHWs feel undervalued but also made them feel less able to do their job properly. In most cases, this had little to do with the technical complexity or training requirements of CHW tasks. Rather, it had to do with whether or not CHWs felt that the health system was providing adequate means and support day to day for them to carry out their tasks. (The important issue of logistical support of supplies and medicines for CHWs is discussed in Paper 10 in this series, on CHW performance and performance assessment [77].) CHWs who felt their work was not feasible because they were not provided the support and resources they needed were demotivated, leaving them feeling ineffective [67]. CHWs also felt great pressure from the people in their communities they were serving to effectively meet their needs. Disappointing fellow community members because their work was not adequately supported was another powerful demotivator, leading to “loss of face” for CHWs in front of the community [67].

In addition to having adequate recognition and support, CHWs were also clearly motivated by tasks that provided them with opportunities for personal growth and professional development (see Finding 5.3). Of course, many more educated CHWs see this work as an opportunity for gaining skills, growing professional networks, and moving up the career/employment ladder (or at least serving as a springboard to a better job with another employer). These are important and well-recognized motivations of many CHWs that also bring their own complications with respect to recruitment and retention [59, 68, 73, 78, 79]. What we are highlighting here, however, is less direct and has to do with the ways in which the day-to-day content of CHW work promotes development in personal, social, or professional dimensions. Work that promotes a sense of autonomy, self-efficacy, and mastery of particular skills or knowledge, or that provides an opportunity to see tangibly the impact of one’s work in the lives of others can be powerfully motivating [61, 69, 80]. Similarly, work that enabled CHWs to master some tasks and move on to other tasks proves motivating, while work that involved the same set of rote tasks month after month with little opportunity for variety or growth are demotivating [73, 81]. These kinds of development opportunities take multiple forms and need not be reduced to a narrow question of professionalization and formal career pathing.

The day-to-day working and social relationships that CHWs have with each other and with other members of the health system also emerged as a central source of CHW motivation. These relationships strongly determine how CHWs experienced their work and interpreted its significance (see Finding 5.4). The relationship between lower-level, health facility-based workers and CHWs is the most central, and often the most problematic, relationship reported in CHW programmes. When this relationship is one of mutual respect and support, CHWs find encouragement and meaning in their work, feel like they are a valuable part of larger team, and are motivated to remain in their jobs and perform well [55, 82]. When these relationships are dysfunctional, however, CHWs can become quickly demotivated [73, 79, 82]. CHWs’ relationships with each other can also be an important source of support and meaning for CHWs. This typically takes the form of informal engagement and encouragement among CHWs, but several studies reported the significant motivating force of formal modes of engagement as well, such as CHW support groups, networks and associations [57, 61, 83, 84].

CHWs also have relationships of social recognition and accountability with a wide range of actors in the health system, in the community, and elsewhere (see Finding 5.5). These relationships are complex and can pull CHWs in opposing directions. CHWs may be managed, for example, by both clinic staff as well as by an NGO that is supporting CHW work (as noted in Paper 1 in this series [1]). They may also be accountable to a community health committee or another community representative body. These actors can have differing, sometimes conflicting, agendas. CHWs caught in the middle can quickly become confused and demotivated. CHW motivation can also be affected if they, for example, gain status and recognition as members of the biomedical health system or are stigmatized for their work on diseases like HIV or TB [68, 79, 85]. They may be seen as less educated, *lay* members of the community by clinic staff, or they may be recognized as valued community representatives [56, 59, 83]. Their social status among these different players is often shifting and complex but typically has a significant impact of CHW motivation.

Finally, it is important to remember that just as incentives and the motivations they shape operate in a richly social environment, those incentives can also affect this social system (see Finding 5.6). Incentives do not simply act on individual CHWs and their internal motivations. They become features of the social and professional system as well. As such, they can take on a range of meanings and can have a range of intended and unintended consequences. The most common example of this described in the literature is the way in which inequitably distributed incentives can produce or deepen social divisions and conflicts in the health system. Providing HIV adherence counsellors with significantly higher stipends than maternal and child health CHWs, for example, can sharpen tensions between these services and lead to an internal brain drain from one to the other. Programmes driven by donor funding (including NGO-funded programmes as well as externally funded vertical disease-specific programmes) can also result in disparities between different health services and programmes as well as local brain drains as CHWs switch their allegiance from one programme to another, and even hearing about such disparities can lead to dissatisfaction and demotivation [83]. Even when issues of equity are not at stake, incentive schemes designed for one set of CHWs can quickly come to be seen as the new standard for CHWs in other parts of the health system. Incentives can send powerful social signals about the meaning and value of particular individuals and services. These signals in turn can shape new relationships and fashion new expectations. The incentive and remuneration scale for CHWs related to the pay grade of the next higher level of health workers can also be an issue even it differences are minimal. The next higher level of workers may resent lesser-trained CHWs receiving benefits that are not that different from their own.

## Impact of broader contextual factors on CHW incentives and motivation

**Table:** 

Key message box 7	
A CHW’s relationship to local cultural contexts, labour markets, the state, and civil society can also greatly shape their motivation	

CHWs, and their motivations, are also affected by the broader contexts in which they work, including local cultural norms and beliefs, local epidemiological priorities, demographic and social structures, social change and conflict, political and ideological histories, relationships to the state and the health system, poverty and inequality, and the nature of labour market, training, and educational opportunities.

When the studies we reviewed cited *context* as a factor in CHW motivation, they usually meant local forms of *culture* and *community*. Indeed, culture and community are powerful contextual forces that can shape CHW motivation, but their meaning is often contested, and their impact is often limited (see Table 6, Finding 6.1). Perhaps the two most powerful sets of cultural practices that shape motivation and behaviour are religion and gender norms. Globally, religious beliefs, while diverse, often provide some kind of rationale or even an explicit duty to perform service to others. These beliefs can trigger and sustain CHW engagement [78, 81, 86]. Similarly, gender norms around care work mean that women are typically seen as *natural* carers, motivating women and demotivating men to engage in (mainly volunteer) CHW work [55, 70, 78, 87]. These beliefs also ensure that women bear the brunt of the care burdens and suffer the bulk of the associated financial, physical, and psychological costs of caring [78, 84].

**Table 6:** The impact of the cultural, political, and economic context on CHW motivation

Findings	Policy and management prompts
6.1 Culture and community are powerful contextual forces that can shape CHW motivation, but their meaning is often contested, and their impact is often more limited than expected	What aspects of local culture (norms, practices, and beliefs) and local community (the social relationships in which one is directly embedded) might have an effect on CHW motivation? Are there, for example, dominant religious values or social pressures to motivate one to serve or to even make sacrifices? How widely are these aspects of local culture really shared? How culturally diverse is the local community? What are the internal lines of division (linguistic, gendered, ethnic, and so forth) that might complicate the prevailing ideas in the community about CHW values and motivations?
6.2 While civil society is an important terrain for the development and support of CHW programmes, its characteristics, and its impact on CHW motivation, also vary considerably from place to place, even within the same country	What is the character of local civil society (e.g., NGOs, community-based organizations, faith-based organizations, and other forms of community organization) and how does civil society engage with CHWs? Is it highly organized or not? Well funded or not? Who runs civil society organizations, and in what sense do they represent broader community interests and perspectives? What is the relationship between civil society and the health system, and how might this relationship, whether positive or negative, affect CHW motivation? What is the level of (dis)trust of civil society in the health system and the government? To what extent does the CHW programme rely on civil society? Is this relationship positive? Is it sustainable?
6.3 The opportunities and barriers embedded in the local labour market define to a large extent how CHWs will interpret the meaning and significance of financial incentives	What is the local economic context like with respect to levels of unemployment, the kinds of jobs available, and the training/skills required, and what are the prospects for further advancement in particular careers? What are the economic opportunities in the local setting that compete most directly for the attention of CHWs, and how many of the CHWs in the programme would be able to take advantage of these opportunities? If these alternative work opportunities either pay more or offer clearer paths for advancement, how do CHWs who remain in their positions explain their staying in place? What role do financial incentives play in this decision?
6.4 The history of a community’s relationship to the health system, and to the state more broadly, shapes what kinds of incentives matter to CHWs and how motivation can be sustained	What is the historical relationship between the local community and the health system and/or state? If it is one of political antagonism and mistrust, how does this impair CHW motivation? If it is one of solidarity and trust, how does this promote CHW motivation? If the health system or state is regarded positively, what kinds of nonfinancial incentives might increase CHWs’ association with the health system/state (e.g., uniforms, ID cards, and so forth)? Alternatively, if the relationship is more conflicted, how can CHWs be shielded from these negative associations? Would incentives that identify CHWs with community relationships/values or that enhance community representation and accountability better motivate CHWs?

Culture and community, however, are always shifting. Gender norms, for example, are shifting in many countries as gendered work and educational opportunities change [78]. Religious beliefs, especially in urban contexts, can vary widely, even among neighbours. Urbanization and modernization are shifting conceptions of individual and community responsibility. Labour migration, local social conflict, and increasing ethnic diversification are making it difficult in many places to identify the boundaries of community or the legitimate representatives of community [55, 78, 82, 83]. The straightforward notion that CHWs can be motivated through reference to local *cultural* frameworks and can be held accountable to and be recognized by a coherent *community* appears less and less tenable in many of the contexts described in these studies, particularly as the recognition of the need to provide CHWs with financial remuneration continues to grow.

Similarly, while civil society is an important terrain for the development and support of CHW programmes, its characteristics, and its impact on CHW motivation, also vary considerably from place to place, even within the same country (see Finding 6.2). Civil society, in the form of community-based organizations and NGOs, may play a role in managing and motivating CHWs, linking them to other community structures and representing them to the state and broader public. The scale and nature of civil society, however, varies considerably globally, as does the degree of organization and available funding. Civil society organizations are also political actors. Their political orientation can have profound effects, positive and negative, on CHW motivation [62, 87].

The shape of the local labour market is another strong factor shaping CHW motivation. Labour market opportunities and barriers define to a large extent how CHWs interpret the meaning and significance of financial incentives (see Finding 6.3). Even though many CHW programmes do not think of themselves as providing formal employment, CHW work is usually seen in relation to the broader labour market. The characteristics of this market—the subsistence/wage labour mix, the patterns of labour migration, the internal class stratification, the presence of alternative work or educational opportunities—all determine how CHWs interpret and respond to, in particular, the financial incentives in their work. In settings where few employment alternatives exist, small financial incentives can be powerful. In settings where alternative options are available, financial incentives will inevitably be compared to potential salaries available from other employers, and there may be high turnover.

Finally, the history of a community’s relationship to the health system and to the state more broadly shapes what kinds of incentives matter to CHWs and how motivation can be sustained (see Finding 5.4). In contexts where the state is mistrusted or citizens feel abused by the state, working for or being associated with the state through the health system can be disincentivizing [87, 88]. Breakdowns in the relationships between community and state can also foreclose opportunities for meaningful consultation and participation in CHW programme decision-making [61, 74]. Like the other contextual factors described in this section, the relationships between state and citizen varies widely, but in many settings, there is an experience of the state as distant and ineffective.

## Designing, evaluating, and readjusting CHW incentive packages

**Table:** 

Key message box 8	
An effective and sustainable approach to CHW motivation requires that CHW programmes are well designed and well managed and that they reflect on and respond to changes in the local context	

While the literature we reviewed paid most of its attention to the initial design of incentive packages, there was also evidence about the need to effectively manage and adjust these packages over time. In many cases, it appears that, once instituted, incentive packages either did not change, or they changed due to external circumstance (e.g., loss of funding) rather than a planned process. The studies do provide enough evidence, however, to conclude that it is critical to see incentives not as a once-off question with a straightforward answer, but as a dynamic process over time that requires attention.

At the individual level, the effects of incentives change over time as do the motivations, needs, and capacities of individual CHWs (see Table 7, Finding 7.1). CHWs of different generations, for example, will bring different political and cultural experiences and expectations to their work [82]. Even for each individual CHW, understanding the effect of the life course is critical since needs and motivations are shaped by a range of life events including marriage, having children, caring for aging parents or family members, critical illness events in families, and educational advancement [69, 72]. CHWs also need variety in their work over the long term, especially if it is repetitive or requires few skills, and they value opportunities for personal and professional development over time (see Table 7, Finding 7.1). Levels of stamina and resistance to physical and emotional burnout also vary with time. Finally, some incentives may have a limited *shelf life* in the sense that they may motivate effectively on initial introduction, but once they are routinized, they can lose their capacity to shape practice [59, 62]. This was the case among some of the CHWs in a TB programme in South Africa who were drawn to this work because of the urgency of the problem and the novelty of this kind of CHW work. Many lost interest over time, however, as they realized that the support to patients would be needed for many years to come, and the government was not likely to provide any payment for this work in the future [60].

**Table 7:** Change over time in CHW motivations

Findings	Policy and management prompts
7.1 At the individual level, the effects of incentives change over time as do the motivations, needs, and capacities of individual CHWs	Have the incentives offered to CHWs remained the same over a long period of time? If so, is there reason to believe that their motivating impact may have lessened? If so, should the incentive be increased, or should it be complemented with other forms of incentives? Do the needs and interests of individual CHWs change significantly over time? As they get older and have families, do they report that previous incentives are less relevant and alternative incentives potentially more effective? If considering varying incentives over time, is there a way to do this that involves sufficient consultation with CHWs as well as equitable and transparent processes and policies for these changes?
7.2 At the programme level, it is important both to understand policy development and programme implementation as a process and to recognize the importance of feedback and participation in the cycle of programme design, implementation, and evaluation	What kind of planning and consultation went into the design of incentives at the beginning of the CHW programme? Were CHWs consulted? If so, how? If not, why not? What do CHWs feel about the current, formal incentive package? What other elements of their work motivate (or demotivate) them to perform and remain in their post? How do they interpret changes in the incentives offered by the programme over time, and how does this shape their expectations about future incentives? Are there opportunities for CHW feedback to be solicited and fed into ongoing management, evaluation, and programme design cycles? Does this consultation process take CHW concerns into account, and do CHWs perceive the process to be fair and responsive?
7.3 At the contextual level, changes in social, cultural, political, economic, health systems, epidemiological, and demographic contexts also affect CHW motivation, and these need to be accounted for in incentive packages	Since the initial design of the CHW programme and its incentives, what has changed in the broader context that might impact on these incentives? Have the priority diseases changed? Have the epidemiological priorities or demographics of the local setting shifted? Have political changes or social conflicts emerged? Have economic opportunities flourished or floundered? Has the health system undergone any significant restructuring that might change how certain services or roles are perceived? Has disease-related stigma abated or intensified?

At the programme level, the studies reviewed have revealed the importance of understanding policy development and programme implementation as a process as well as the importance of involving CHWs in feedback and participation in the cycle of programme design, implementation, and evaluation (see Table 7, Finding 7.2). Rather than incentives being a static feature of a CHW programme that are settled on at the outset and then left unchanged, the most successful volunteer CHW programmes appear to have initially developed incentive packages through a process of consultation and refinement and then sustained the impact of these incentives by continuing to engage CHWs in their evaluation and reformulation [62, 65, 89]. CHWs seem to recognize and accept the realities of resource constraints in these settings and the process-oriented nature of health programmes and policies. What is demotivating to them is being left out of the process and/or watching the process stall midstream [24].

Finally, at the contextual level, changes in social, cultural, political, economic, health systems, epidemiological, and demographic contexts also impact CHW motivation and need to be accounted for (see Table 7, Finding 7.3). New economic opportunities, emerging critical health challenges, shifting cultural norms, improvements in access to healthcare, changing relationships to the state all change the meaning and context of CHW work and have an impact on what kinds of incentives might motivate CHWs to join programmes or to remain in their posts as CHWs and perform well [57, 81, 86]. Most of these changes are slow, but some, such as the emergence of the HIV or the COVID-19 pandemic, transition to new governments, or a global economic crisis, can happen quite suddenly.

## WHO Recommendations for CHW reimbursement and contracts

**Table:** 

Key message box 9	
Recent WHO guidelines have emphasized the importance of fair labour practices, supportive work environments, and attention to principles of gender equity in CHW programmes	

In 2018 WHO released its first ever set of guidelines for optimizing health system and policy support for optimizing the contribution of CHWs [15, 43]. One of the 15 recommendations concerned remuneration, and another concerned contractual agreements. WHO recommends that practicing CHWs receive a financial package “commensurate with the job demands, complexity, number of hours, training and roles that they undertake” [p. 47]. WHO also recommends that paid CHWs be provided with a “written agreement specifying role and responsibilities, working conditions, remuneration and workers’ rights”. These two recommendations comprised two of only three strong recommendations in the set of 15. The other strong recommendation concerned the importance of engaging communities in the planning, selection, and oversight of CHWs.

As indicated before, it also recommended that CHWs not be paid exclusively or predominantly according to performance-based incentives because of the likelihood of CHWs neglecting responsibilities that were not incentivized.

The WHO guidelines expressed concern that reliance on voluntary CHWs is “inconsistent with the international agenda on decent work and particularly with Sustainable Development Goal (SDG) 8, promoting decent work and economic growth”. This recommendation was bolstered by the fact that continued reliance on voluntary work from CHWs could perpetuate gender disparities in access to employment and income opportunities and be inconsistent with SDG 5, achieving gender equality and empowerment of all women and girls. The WHO guidelines did not rule out the use of volunteer CHWs, but it did express concern about the use of volunteer CHWs who did not have any other source of livelihood.

The Guideline Development Group noted the importance of nonmonetary incentives, but did not consider them as a substitute for financial remuneration, nor did it consider remuneration a substitute for nonfinancial incentives. A conducive and respectful work environment and opportunities for professional development and career advancement are all important to the package of financial and nonfinancial incentives.

CHW roles and identities have lacked clarity because CHWs work at the interface between community and health systems. The presence of a formal contract that clearly specifies the roles and responsibilities of the CHW, the community, and the health system, specifying working conditions and rights, job responsibilities, duration of employment, and remuneration terms is recommended. Such contracts serve as an incentive and contribute to job stability and security, enhance occupational protection and safety, and set the stage for professional development opportunities. A formal contract also provides a basis for the health system to hold CHWs accountable for their work. Such contracts are not needed for volunteer CHWs according to the guidelines.

## Conclusion

This paper has described the range and types of incentives offered to CHWs and the underlying factors affecting CHW motivation in a number of different domains, including the ways incentives work at the individual level, the role of the health system and community context, the impact of broader cultural, political, and economic realities on CHW incentives and motivation, and the importance of approaching incentive strategies and motivation as a process that unfolds over time and needs to be managed as such.

We have also marked out for special attention the role of change over time in understanding CHW incentives and motivations. Time matters not only for thinking how motivations might change over time, but also how incentives may shift in their ability to incentivize as time passes. Change over time is also a critical planning and management perspective and tool, one that works against the overly static models of human behaviour and motivation that we found in many studies. This is especially critical for CHW programmes since, unlike most other healthcare workers, CHWs tend to lack institutional, professional, or disciplinary frameworks that could bring some stability and consistency over time to the standards and practices of their work.

This review has also highlighted the critical role of individual experience, local context, and the social construction of incentives in producing and sustaining motivation. Many of the findings above point to the fact that there is no easy one-to-one relationship among incentives, motivation, and practice. Straightforward functionalist or behaviourist theories of motivation tend to assume this kind of linearity, but the review has identified the thorough-going importance of relationships, contexts, and day-to-day lived experience for understanding how and why incentives might work in a particular place and time.

This review adds to the existing literature on CHW incentives in a number of other ways as well. We have attempted to distil out a number of interrelated findings that contribute to a “middle-range theory” [90] of CHW incentives and motivation, findings that synthesize the findings of many smaller underlying studies in an effort to identify patterns and dynamics that, while not *universal* or *natural*, are common across a variety of settings and CHW programmes. In doing so, we hope to have complicated some of the more simplistic theoretical assumptions of CHW incentives and motivation (e.g., a false binary between altruistic and material motives), while also pointing to empirical and theoretical gaps in the literature.

We have also highlighted the important role of indirect, system-level features of the health system and/or broader context for CHW motivations, aspects that often cannot be directly acted on by individual CHWs or their programme managers and designers. There are two reasons that practitioners and policy-makers should nonetheless pay special attention to these domains. Firstly, the “stick” factors in CHW work—the factors that keep one in a job—may be weak [91]. This means that careful attention to all the factors that might improve CHW retention and motivation is critical. Secondly, managers and policy-makers at the programme level often do have broad discretion when it comes to designing and implementing programmes in ways that reflect and respond to the local contexts. Understanding and anticipating how these more upstream factors may shape motivation at the local level is a critical aspect of designing and managing effective and responsive incentive schemes.

Another contribution of this review has been to develop a range of *policy and management prompts* linked to each key review finding (see Tables 3, 4, 5, 6 above) for programme managers and policy-makers to consider when planning and implementing CHW interventions. These prompts have been informed by evidence in the studies we reviewed, but they also reflect our own assessment of how these findings might be usefully translated into key questions to consider for policy-makers and programme managers. Rather than develop more conventional *recommendations* (that would likely only apply in certain in contexts), we have offered prompts to guide reflection and further action, questions that take into account the key lessons learned from the studies we reviewed. In Table 8 we have also provided an abridged version of these prompts that policy-makers and programme managers could quickly use to guide their deliberations.

**Table 8:** Summary of key prompts for policy-makers and managers

Theme	Key questions to consider
Incentives at the individual level	
Managing the relationship between altruism and CHWs’ basic survival needs	What altruistic motivations keep CHWs engaged in their work? How does the CHW programme reinforce or undermine these sources of altruism? Are there tensions between CHWs’ altruistic motives and their need to provide for their basic material needs? How does the CHW programme balance these different motives?
Other extrinsic motivations besides salary	How can your programme address some of CHWs other material needs like training and certification opportunities, referrals for/preferential access to health, welfare, housing, or educational benefits, food parcels, transport money, airtime, etc.? Can you provide these material benefits in a way that is socially acceptable and avoids breeding competition or conflict
Identifying and maintaining intrinsic motivation	How can your programme identify and support intrinsic CHW motivations, such as a personal connection to the health problem addressed or some other altruistic motivation?
Incentives within the health system	
Valuing CHW team members and clarifying roles and responsibilities	In what ways are CHWs recognized for their contributions to the health system? Do CHWs have clear job descriptions and distinct roles in the settings where they work? Do others clearly understand their roles and responsibilities
Ensuring CHW work feels “do-able”	How are CHW responsibilities decided on, and how do managers ensure CHWs have the capacity to fulfil them? Do CHWs have the opportunity to speak about issues of workload or technical complexity? Can CHWs or local programme managers reorganize tasks to improve the “do-ability” of the role?
Supporting personal growth and professional development	What elements of the CHW role promote personal growth (e.g., social, emotional, psychological, or intellectual development)? What elements of the CHW role promote basic professional development (e.g., computer, administrative, financial, or logistical skills)? How can these elements be strengthened in the programme?
Supporting CHWs’ working and social relationships with others	Do CHWs ever get the chance to work with or interact with each other in their daily work? Are there formal or informal opportunities for CHWs to spend time with each other outside of the context of daily work? How could the relationships between CHWs and healthcare professionals be improved?
Effective forms of recognition and accountability for CHWs	Are there processes for recognizing service and dedication among CHWs by the health system, the community at large, or other stakeholders? How are conflicts or issues of poor performance among CHWs handled and by whom? Can this be improved?
Managing the social impact of CHW incentives	Could CHW incentives introduce new dynamics into CHW social relationships such as competition, status conflicts, or other forms of division? How can incentives be designed and managed in a way that limits any negative effects on these social relationships?
Incentives in cultural, political and economic context	
Culture and community in CHW programmes	What aspects of the local cultural and community context might increase or reduce CHW motivation? What are the potential lines of division within a community—linguistic, gendered, ethnic, and so forth—that might complicate CHW experiences and motivations?
Complex engagements with civil society	What is the character of the local civil society and how does civil society engage with CHWs? What is the relationship between civil society and the health system, and how might this relationship, whether positive or negative, affect CHW motivation?
CHW incentives in the context of local labour conditions	What is the local economic context like, and what economic opportunities compete most directly for the attention of CHWs? If these alternative work opportunities either pay more or offer clearer paths for advancement, how does this affect CHW motivation?
CHW incentives and their relationships to the government	Are the relationships between the local community and the health system and/or state positive or negative, and how does this affect CHW motivation? What kinds of incentives might increase CHW’s motivation by building on a positive relationship, or shielding a CHW from a negative relationship?
Changes over time in CHW motivation	
Managing the changing impact of incentives over time	Has the motivating impact of CHW incentives lessened over time, and if so, how can the incentive package be adjusted? As CHWs get older and have families, are previous incentives less relevant, and are alternative incentives potentially more effective?
Approaching the CHW policy cycle as an ongoing process	What kind of planning and consultation went into the design of incentives at the beginning of the CHW programme? What do CHWs feel about the current package of incentives? Are there opportunities for meaningful CHW feedback to be solicited and fed into ongoing management, evaluation, and programme design cycles?
Matching incentives to changes in social, cultural, political, economic, health systems, and demographic contexts	Since the initial design of the CHW programme and its incentives, what has changed in the broader social, political, economic, or health systems context that might impact on these incentives? How should the incentive package be adjusted in response to these changes?

The tables link individual review findings to specific policy prompts, but a number of broader concluding principles also emerge out of these findings. These include: the importance of ongoing engagement between CHWs and managers (in the form of supervision, feedback, review, and consultation); the need to give local-level managers flexibility in designing and sustaining appropriate incentive options over time; and the complexity and nonlinearity of the task of incentivizing CHWs. While the great diversity of local contexts and constraints prevents us from making recommendations that are any more specific than this, we do believe that CHW programmes that take seriously (1) the need to effectively support CHWs in ways that are (2) meaningfully responsive to local conditions and (3) more holistic in their approach will ultimately enjoy greater CHW motivation and impact.

A final concluding point is important in understanding why efforts to incentivize CHWs seem to so often struggle over time. It is clear that many of the best practices for incentivizing CHWs to apply, remain in, and do well at their work require an effective and responsive health system. Many of the most critical incentivizing and disincentivizing factors are, in fact, within the scope of the health system to affect in some way. However, it is most often in the settings with the weakest health systems that CHWs are relied upon to provide coverage for critical gaps in care. This is an old and familiar paradox to those researching CHW programmes [61, 74, 76, 86], but it bears repeating in this context. Using CHWs to improve the health outcomes of communities requires much more than just instructing them to provide a specific technical intervention in the absence of a supportive and enabling environment, and hoping their input will fix deeper problems in the system. Using CHWs effectively involves the much more difficult work of building and sustaining a meaningful, responsive, well-resourced, and well-managed role for CHWs as part of both the health system as well as the broader communities and contexts in which they work.

## Data Availability

All of the data for this paper came from published literature cited in the reference list and are publicly available.

## Abbreviations

CHEW: Community health extension worker
CHW: Community health worker
NGO: Nongovernmental organization
TB: Tuberculosis

## Footnotes

1 In a realist assessment, special consideration is given to the sociohistorical context in which the intervention(s) undergoing assessment was/were implemented.
